# Altered gyrification in chemotherapy‐treated older long‐term breast cancer survivors

**DOI:** 10.1002/brb3.3634

**Published:** 2024-08-21

**Authors:** Ebenezer Daniel, Frank Deng, Sunita K. Patel, Mina S. Sedrak, Heeyoung Kim, Marianne Razavi, Can‐Lan Sun, James C. Root, Tim A. Ahles, William Dale, Bihong T. Chen

**Affiliations:** ^1^ Department of Diagnostic Radiology City of Hope National Medical Center Duarte California USA; ^2^ Department of Population Science City of Hope National Medical Center Duarte California USA; ^3^ Department of Medical Oncology City of Hope National Medical Center Duarte California USA; ^4^ Center for Cancer and Aging City of Hope National Medical Center Duarte California USA; ^5^ Department of Supportive Care Medicine City of Hope National Medical Center Duarte California USA; ^6^ Neurocognitive Research Lab Memorial Sloan Kettering Cancer Center New York New York USA

**Keywords:** breast cancer, cancer‐related cognitive impairment, chemotherapy, gyrification index

## Abstract

**Purpose:**

The purpose of this prospective longitudinal study was to evaluate the changes in brain surface gyrification in older long‐term breast cancer survivors 5–15 years after chemotherapy treatment.

**Methods:**

Older breast cancer survivors aged ≥ 65 years treated with chemotherapy (C+) or without chemotherapy (C‐) 5–15 years prior and age‐ and sex‐matched healthy controls (HC) were recruited (time point 1 (TP1)) and followed up for 2 years (time point 2 (TP2)). Study assessments for both time points included neuropsychological (NP) testing with the NIH Toolbox cognition battery and cortical gyrification analysis based on brain MRI.

**Results:**

The study cohort with data for both TP1 and TP2 consisted of the following: 10 participants for the C+ group, 12 participants for the C‐ group, and 13 participants for the HC group. The C+ group had increased gyrification in six local gyral regions including the right fusiform, paracentral, precuneus, superior, middle temporal gyri and left pars opercularis gyrus, and it had decreased gyrification in two local gyral regions from TP1 to TP2 (*p *< .05, Bonferroni corrected). The C‐ and HC groups showed decreased gyrification only (*p *< .05, Bonferroni corrected). In the C+ group, changes in right paracentral gyrification and crystalized composite scores were negatively correlated (*R *= –0.76, *p *= .01).

**Conclusions:**

Altered gyrification could be the neural correlate of cognitive changes in older chemotherapy‐treated long‐term breast cancer survivors.

## Graphical abstract

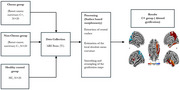

## 1 INTRODUCTION

More than 4 million women have a history of breast cancer, and additional newly identified 287,850 cases have been reported as of January 1, 2022 in the United States alone (Miller et al., 2022). Besides, more than 2.7 million breast cancer survivors are 65 years and older (Miller et al., 2022). Prior studies have shown that chemotherapy‐treated breast cancer survivors suffer from cancer‐related cognitive impairment (CRCI) (Calvio et al., 2010; Lyon et al., 2016; Mandelblatt et al., 2014). CRCI mainly affects memory, attention, and executive functioning in older long‐term survivors (Joly et al., 2015; Országhová et al., 2021; Pendergrass et al., 2018).

Neuroimaging studies have shed light on brain structural and functional alterations underlying CRCI in breast cancer survivors (Daniel et al., 2022; Sousa et al., 2020; Wefel et al., 2015). Previous studies have found a significant reduction in brain gray matter (GM) and white matter in long‐term breast cancer survivors at ten or even 20 years after chemotherapy (de Ruiter et al., 2012; Koppelmans et al., 2014; Stouten‐Kemperman et al., 2015). GM atrophy has been known to have a significant association with cognitive dysfunction amongst breast cancer survivors (Chen et al., 2018; Hosseini et al., 2012; Kesler et al., 2017; Lepage et al., 2014; Li et al., 2018; McDonald et al., 2010; Perrier et al., 2020). Our previous study of older breast cancer survivors showed cortical thinning in older long‐term breast cancer survivors (Daniel et al., 2022).

Cortical Gyrification is a morphometric feature related to the geometry of the brain surface (Cao et al., 2017; Luders et al., 2012). Since GM forms an outer layer of the brain surface, the alterations in gyrification result in changes in cortical surface area and cortical GM volume (White et al., 2010). Gyrification analysis focuses on brain morphometric features that are not identified by GM or cortical thickness (Spalthoff et al., 2018). During brain development, gyrification increases and peaks during childhood, promptly decreases during the adolescent stage and then gradually decreases with age (Gregory et al., 2016; Madan, 2021; Spalthoff et al., 2018; van Haren et al., 2011). Thus, gyrification is expected to decrease with aging (Spalthoff et al., 2018). The decreased gyrification is considered an early morphometric biomarker for cognitive changes in patients with Alzheimer's disease (AD) (Liu et al., 2012), subjective cognitive impairment (Youn et al., 2021), autism (Schaer et al., 2013), mild traumatic brain injury (Gharehgazlou et al., 2022) and in healthy individuals with normal aging (Lamballais et al., 2020). In addition to the decreased gyrification patterns, prior studies on schizophrenia (Spalthoff et al., 2018), AD (Núñez et al., 2020), traumatic brain injury (Wilde et al., 2021) and autism (Yang et al., 2016) have also showed increased gyrification patterns which was associated with cognitive impairment. A previous study of CRCI showed decreased gyrification in patients with breast cancer aged 29 to 68 years shortly after chemotherapy (Zhou et al., 2022). However, there is limited literature on gyrification in older long‐term breast cancer survivors.

Here, we conducted a longitudinal study to assess the brain surface gyrification changes in older breast cancer survivors. We hypothesized that gyrification would be decreased in the older long‐term breast cancer survivors with exposure to chemotherapy, which would be correlated with cognitive changes. To test this hypothesis, we assessed brain gyrification on brain MRI and cognitive performance via neuropsychological (NP) testing in older breast cancer survivors who had chemotherapy treatment 5−15 years prior to enrollment and compared this group to the two control groups including the no‐chemotherapy group and the healthy control group over two years.

## 2 METHODS

### 2.1 Study participants

The study was a neuroimaging substudy of a multicenter trial of long‐term breast cancer survivors (parent trial: Cognition in Older Breast Cancer Survivors: Treatment Exposure, APOE and Smoking History, NCT02122107). Breast cancer survivors treated with chemotherapy (C+) or without chemotherapy (C‐) 5–15 years prior and age‐ and sex‐matched healthy controls (HC) with no history of cancer were enrolled. All participants were aged ≥ 65 years at the time of initial enrollment. Study assessment included brain MRI and NP attesting with the National Institute of Health (NIH) Toolbox Cognition Battery both at time point 1 (TP1) upon enrollment and at the 2‐year interval at time point 2 (TP2). The eligibility criteria for breast cancer survivors were the following: woman aged 65 years and older with a history of stages I–III breast cancer with or without chemotherapy treatment at 5–15 years after surviving breast cancer, and no contraindications such as orbital metal or claustrophobia for brain MRI scans. Exclusion criteria included the following: history of stroke, psychiatric disease, metastatic disease, or any other cancer. Age‐ and sex‐matched HCs were enrolled with similar criteria except for the history of cancer. The HCs were recruited via local newspaper advertisements, patient referrals, and community health fairs.

The Medical Outcomes Study (MOS) Physical Health Scale questionnaire data was obtained from all participants during the study. The MOS Physical Health Scale measured a broad range of physical functioning, with 10 questions ranging from “Can you bathe and dress yourself?” to “Can you perform vigorous activities, such as running or lifting heavy objects?” Items were rated on a 3‐point Likert scale measuring independence in performing the activity. The summary score ranges from 0 to 100 with higher score being better physical health function (Stewart & Ware, 1992). In addition, the Center for Epidemiologic Studies Depression Scale (CESDS) questionnaire data was also obtained from all participants, which contained life style data. The CESDS questionnaire consisted of 20 questions designed to measure mood and depressive symptoms over the past week. Scores on the CESDS ranged from 0 to 60, with a threshold of 16 or higher indicating significant distress (Siddaway et al., 2017). Questions covered a wide range of mood and behavior aspects, including feelings of sadness, hopelessness, changes in appetite, concentration difficulties, sleep quality, and social interactions.

This study was approved by the Institutional Review Board (IRB) of City of Hope National Medical Center. Written informed consent was obtained from all participants in compliance with institutional guidelines and the Declaration of Helsinki, as well as local, state, and federal regulations from all participating subjects.

### 2.2 MRI acquisition and gyrification analysis

All brain MRI scans were acquired using the same in‐house 3T VERIO Siemens scanner (Siemens, Erlangen, Germany). Structural three‐dimensional (3D) T1‐weighted magnetization prepared rapid gradient echo (MPRAGE) images were acquired with the following parameters: TR = 1900 ms; TE = 2.94 ms; inversion time = 900 ms; FA = 9°; and voxel size = 0.45 × 0.45 × 1.5 mm^3^. Incidental brain pathology was assessed on the T1‐weighted MPRAGE and fluid‐attenuated inversion recovery (FLAIR) images by the neuroradiologist in the study (BC). The cortical gyrification analysis was performed using the Computational Anatomy Toolbox (CAT12) (Spalthoff et al., 2018) from the T1‐weighted images. All images were manually re‐oriented using the statistical parametric mapping toolbox (version SPM12) (Wellcome Department of Cognitive Neurology, UK). The CAT12 and SPM12 toolboxes for our analysis were based on MATLAB (R2019b). The mean gyrification values were analyzed based on Desikan‐Killiany (DK40) cortical atlas (Chaudhary et al., 2020; Desikan et al., 2006). We followed the standard pipeline and settings for preprocessing and gyrification analysis (Spalthoff et al., 2018). The main steps were as follows: (i) extraction of central surface; (ii) estimation of the local absolute mean curvature from each vertex point within the 3 mm of this central surface given point; (iii) smoothing and resampling of the gyrification maps using full width at half maximum (FWHM) Gaussian filter at 20 mm.

### 2.3 NP testing with NIH toolbox for cognition

The NP testing was performed using the NIH Toolbox Cognition Battery (Gershon et al., 2013; Weintraub et al., 2013). This cognitive testing battery generated seven individual scores for List Sorting Working Memory, Picture Sequence Memory, Pattern Comparison Processing Speed, Oral Reading Recognition, Picture Vocabulary, Flanker Inhibitory Control, and Dimensional Change Card Sorting. Additionally, the crystalized, fluid, and total composite cognition scores were also generated.

### 2.4 Statistical analysis

Clinical and demographic information was assessed using analysis of variance (ANOVA) for continuous variables. Categorical variables were analyzed using Fisher's exact tests. Threshold of *p‐*value at .05 was considered statistically significant for both continuous and categorical variables, and all tests were two‐sided.

NP test performance was analyzed using a generalized linear model (GLM) with the correlation of repeated measurements within subjects (Daniel et al., 2022; Laird & Ware, 1982). Group (C+; C‐; HC) and time‐point (TP1; TP2) were considered categorical fixed effects in this analysis. Using the GLM, we tested the following: (1) whether there were any differences in NP scores between the three groups at TP1 or TP2; (2) whether there were any significant longitudinal differences within the group; (3) whether there was a group by time interaction effect. SAS 9.3 (SAS Institute, Cary, NC) was used for data analyses.

Whole brain surface gyrification was compared between groups at TP1 using two‐sample *t*‐test. Within group, longitudinal change over the 2‐year study interval was tested using paired *t*‐tests. In both analyses, effects were corrected for multiple comparisons for the whole brain using Bonferroni correction in the CAT12 software with a significance threshold of *p *< .05. The correlations of the mean gyrification values with the NP composite scores were tested using linear regression analysis with a *p* of .05 being considered significant. The linear regression analysis and group by time interaction were tested using the statistical package for the social science software (SPSS, v 27, Chicago, IL).

## 3 RESULTS

### 3.1 Demographic data

At TP1, a total of 60 participants were enrolled with 20 participants for each of the three groups, that is, the C+, C‐, and HC groups. At TP2, due to attrition from loss to follow‐up, new cancer, new memory problems, refusal to continue with the study and death, the final cohort consisted of 10 participants for the C+ group, 12 participants for the C‐ group, and 13 participants for HC group (Daniel et al., 2022). There were no significant differences among the groups in age (*p *= .75), education (*p *= .80), or race (*p *= .37) (Table 1). More detailed clinical and demographic information for this cohort has been reported in our prior study of cortical thickness (Daniel et al., 2022). In the C+ group, 80% of survivors had Stage II breast cancer. The C‐ group consisted of 50% survivors in stage 0, 33% survivors in stage I and 17% survivors in stage II. In the C+ group, 90% of survivors had treatment with non‐trastuzumab regimen and 10% of survivors with trastuzumab regimen (Table 1). The chemotherapy treatment duration data were available for 7 out of 10 patients who consisted of the C+ group with both TP1 and TP2 imaging data for the analysis. These seven patients had the mean duration of chemotherapy treatment from start to end of 103.71 days (SD = 37.79), with a range of treatment days spanning from 54 to 154 days. The total dose of chemotherapy for the patients in the C+ group was not available in the database.

**TABLE 1:** Demographic and clinical information.

Parameters	C+*N* = 10	C‐*N* = 12	HC *N* = 13	*p*
**Age** years				
Mean (SD)	74.70 (5.44)	76.50 (4.28)	75.54 (6.63)	.752
Median (range)	72.5 (68–84)	75.5 (71–86)	75.00 (67–88)	
**Race** (*N*, %)				
White or Caucasian	8 (80)	11 (92)	13 (100)	.373
Black	1 (10)	1 (8)	.	
Asian, Native Hawaiian	1 (10)	.	.	
Other	.	.	.	
**Highest grade** (*N*, %)				
High school or less	2 (20)	4 (33)	4 (31)	.805
College or above	8 (80)	8 (67)	9 (69)	
**AJCC Stage** (*N*, %)				
DCIS	1 (10)	6 (50)	.	
Stage I	1 (10)	4 (33)	.	
Stage II	8 (80)	2 (17)	.	
**Regimen** Non‐trastuzumab regimen (*N*, %)	9 (90)			
Trastuzumab regimen (*N*, %)	1 (10)			

*Note*: *N* = number of subjects. For all the above comparisons, ANOVA or Fisher tests were used (for continuous or categorical data, respectively). Parameters were significant with threshold at *p* of .05.

**Abbreviations**: AJCC: American Joint Committee on Cancer; BMI: body mass index; C‐: no‐chemotherapy group; C+: chemotherapy group; DCIS: ductal carcinoma in situ; HC: healthy control group; SD: standard deviation; TP1: time point 1; TP2: time point 2.

The physical activity scores for each group were calculated using MOS Physical Health Scale questionnaires, with the C+ group having a mean value of 78.46 (SD = 25.70), the C‐ group with a mean value of 85.00 (SD = 16.79), and the HC group with a mean value of 84.67 (SD = 15.64) at time point TP1. At TP 2, the C+ group had a mean value of 74.5 (SD = 26.92), the C‐ group had a mean value of 80.5 (SD = 22.66), and the HC group had a mean value of 81.67 (SD = 14.03). The statistical analysis using a mixed model with compound symmetry covariance showed no significant differences in the MOS Physical Health Scale data in time (*p *= .05), group (*p *= .866) or the group by time interaction (*p *= .748). The statistical analysis showed no significant differences between groups in the CESDS scores for depression screening (*p *= .552) at time point 1 (TP 1) with the C+ group scored at 8.40 with SD of 8.24, the C‐ group scored at 6.00 with SD of 6.49, and the HC group scored at 6.65 with SD of 6.62. No statistically significant differences in the CESDS scores were noted longitudinally between TP1 and TP2 (*p *= .359).

### 3.2 Gyrification data

There were no significant gyrification differences at TP1 between C+ versus C‐, C+ versus HC, and C‐ versus HC group (*p* > .05, Bonferroni corrected).

**FIGURE 1:**
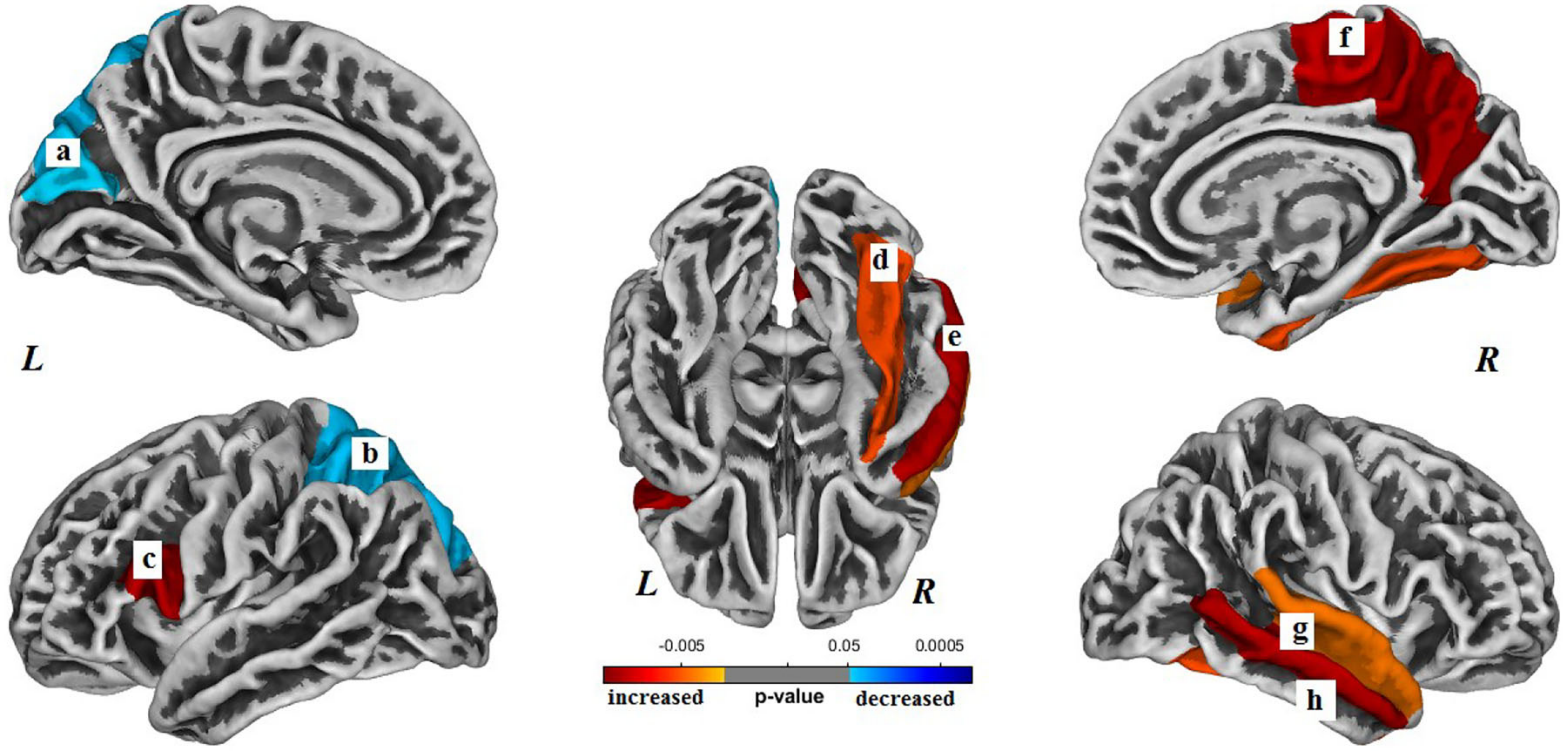
Brain regions with longitudinal changes in gyrification within the chemotherapy (C+) group. The altered regions are (a) left cuneus, (b) left superior parietal gyrus, (c) left pars opercularis, (d) right fusiform gyrus, (e) right middle temporal gyrus, (f) right precuneus, (g) right superior temporal gyrus, and (h) right middle temporal gyrus. *L*, left hemisphere; *R*, right hemisphere. Results were Bonferroni corrected at significant level of .05.

Within the C+ group, gyrification was significantly increased in six regions and decreased in two regions longitudinally over the 2‐year study interval (*p *< .05, Bonferroni corrected) (Table 2). The brain regions with increased surface gyrification in the C+ group included the following (Bonferroni corrected): left pars opercularis gyrus (*p *< .001), right superior temporal gyrus (*p *< .001), right middle temporal gyrus (*p *< .001), right precuneus gyrus (*p *< .001), right paracentral gyrus (*p *< .001), and right fusiform gyrus (*p *= .004) (Figure 1). The brain regions with decreased surface gyrification in the C+ group included the following (Bonferroni corrected): left superior parietal gyrus (*p *= .030) and left cuneus gyrus (*p *= .030).

**TABLE 2:** Gyrification results.

Changes	Size (vertexes)	*p*‐Value (corrected)	Overlap of atlas region (%)	Brain region (DK40)
**C+ group**:				
TP2 > TP1	3119	.00034	100	Left pars opercularis
	11,925	.00034	58	Right superior temporal
			42	Right middle temporal
	11,806	.00025	68	Right precuneus
			32	Right paracentral
	4661	.00435	100	Right fusiform
TP2 < TP1	12,086	.03061	87	Left superior parietal
			13	Left cuneus
**C‐ group**:				
TP2 < TP1	11,450	.00001	41	Left fusiform
			37	Left lingual
			22	Left isthmus cingulate
	8600	.00105	100	Left paravaginal
	4351	.00616	100	Right lateral orbitofrontal
	4198	.0010	100	Right inferior temporal
	3494	.00021	100	Right caudal middle frontal
**HC group**:				
TP2 < TP1	33,739	.00095	36	Left superior frontal
			28	Left postcentral
			22	Left precuneus
			10	Left paracentral
			4	Left caudal anterior cingulate
	1064	.00022	100	Left transverse temporal
	15,372	.00040	77	Right superior frontal
			23	Right caudal middle frontal
	8150	.00019	100	Right supramarginal

*Note*: Cluster size > 10. Results were significant with threshold at *p* of .05.

**Abbreviations**: HC: healthy control group; TP1: time point 1; TP2: time point 2; C‐: no‐chemotherapy group; C+: chemotherapy group; DK40: Desikan atlas.

**FIGURE 2:**
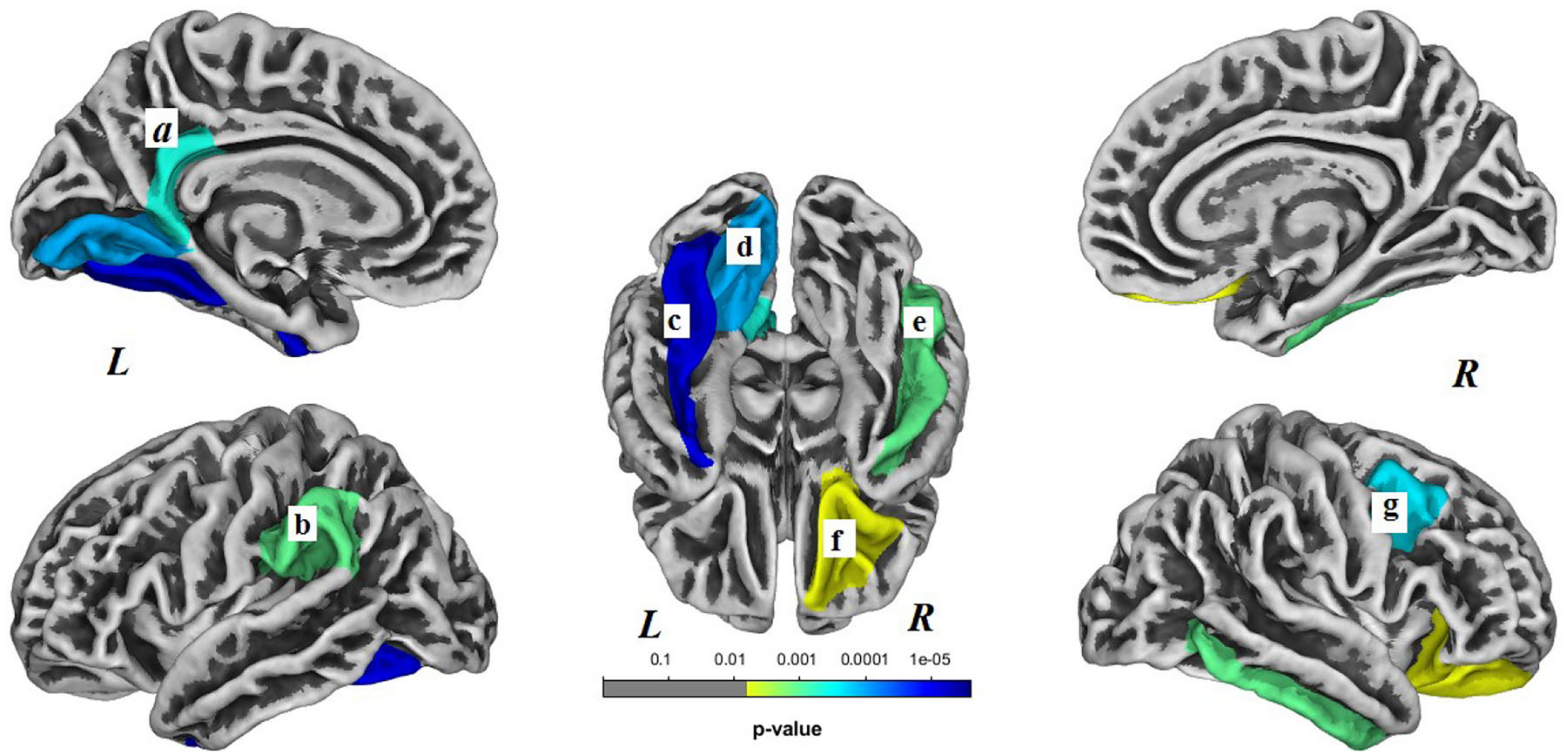
Brain regions with decreased gyrification within the nonchemotherapy control (C‐) group. These regions included the following: (a) left isthmus cingulate gyrus, (b) left supramarginal gyrus, (c) left fusiform gyrus, (d) left lingual gyrus, (e) right inferior temporal gyrus, (f) right lateral orbitofrontal gyrus, (g) right caudal middle frontal gyrus. *L*, left hemisphere; *R*, right hemisphere. Results were Bonferroni corrected at significant level of .05.

Within the C‐ group, brain surface gyrification was significantly decreased in seven regions (*p *< .05, Bonferroni corrected) and no regions showed increased gyrification. Decreased surface gyrification within the C‐ group was noted in left fusiform gyrus (*p *< .001), left lingual gyrus (*p *< .001), left isthmus cingulate gyrus (*p *< .001), left supramarginal gyrus (*p *= .001), right lateral orbitofrontal gyrus (*p *= .006), right inferior temporal gyrus (*p *= .001), and right caudal middle frontal gyrus (*p *< .001) (Figure 2).

**FIGURE 3:**
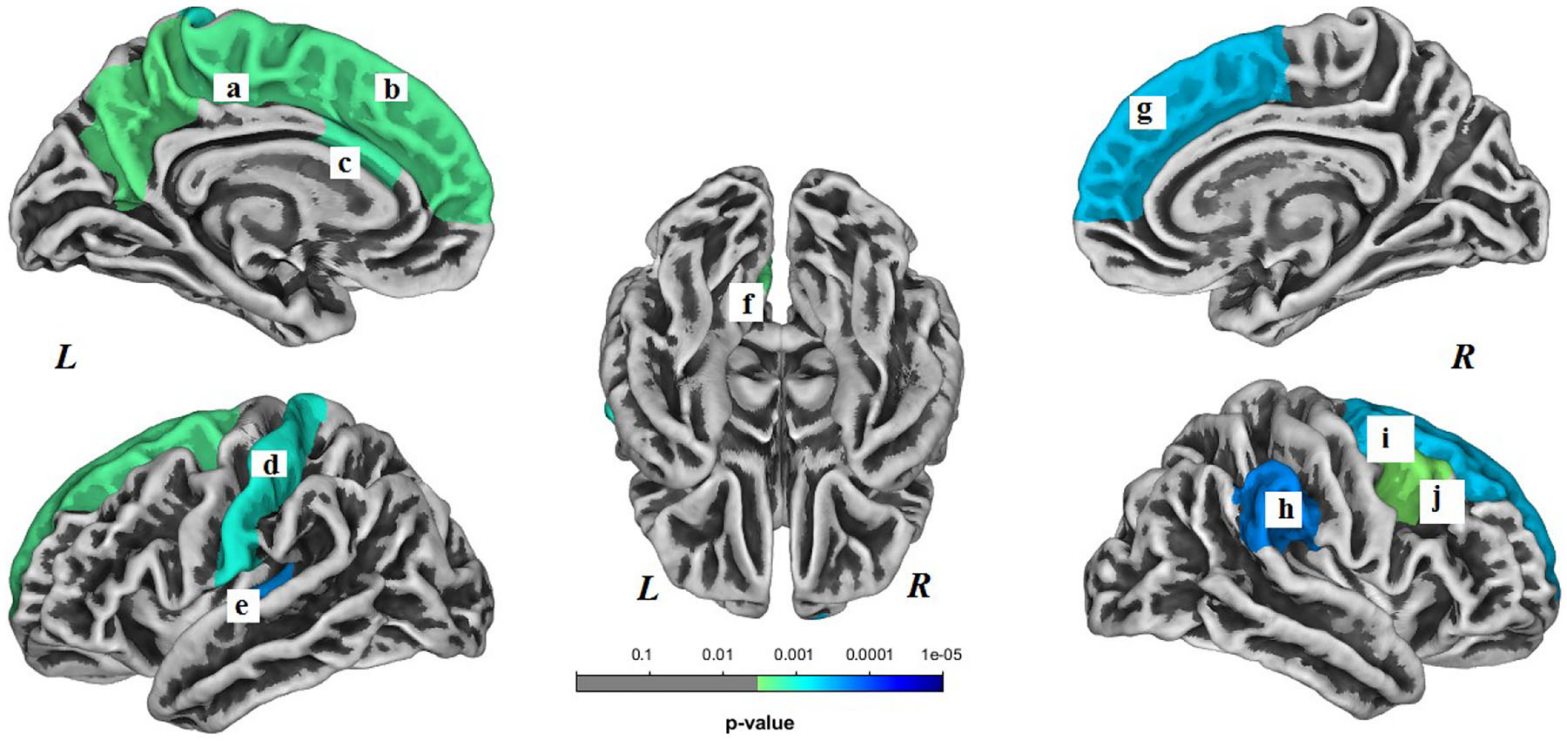
Brain regions with decreased gyrification within the healthy control (HC) group. These regions included the following: (a) left paracentral gyrus, (b) left superior frontal gyrus, (c) left caudal anterior cingulate gyrus, (d) left postcentral gyrus, (e) left transverse temporal gyrus, (f) left precuneus, (g) right superior frontal gyrus, (h) right supramarginal gyrus, (i) right superior frontal gyrus, and (j) right caudal middle frontal gyrus. *L*, left hemisphere; *R*, right hemisphere. Results were Bonferroni corrected at significant level of .05.

In the HC group, brain surface gyrification was significantly decreased in nine regions (*p *< .05, Bonferroni corrected) and no regions showed increased gyrification longitudinally. Decreased brain surface gyrification was noted in the following regions: left superior frontal gyrus (*p *< .001), left postcentral gyrus (*p *< .001), left precuneus gyrus (*p *< .001), left paracentral gyrus (*p *< .001), left caudal anterior cingulate gyrus (*p *< .001), left transverse temporal gyrus (*p *< .001), right superior frontal gyrus (*p *< .001), right caudal middle frontal gyrus (*p *< .001), and right supramarginal gyrus (*p *< .001) (Figure 3).

There was no significant gyrification difference noted in group‐by‐time interaction analysis (*p* > .05, Bonferroni corrected).

### 3.3 NP testing scores

The detailed NP score results of the NIH Toolbox cognition battery testing scores have been reported in our prior study of cortical thickness (Daniel et al., 2022). Briefly, the C+ group showed significantly decreased total composite score (*p *= .01), fluid composite score (*p *= .03) and picture vocabulary score (*p *= .04) across the 2‐year interval. No significant changes in NP scores were noted in the C‐ and HC group at a threshold of *p* values at .05.

### 3.4 Correlation between gyrification data and the NP scores

**FIGURE 4:**
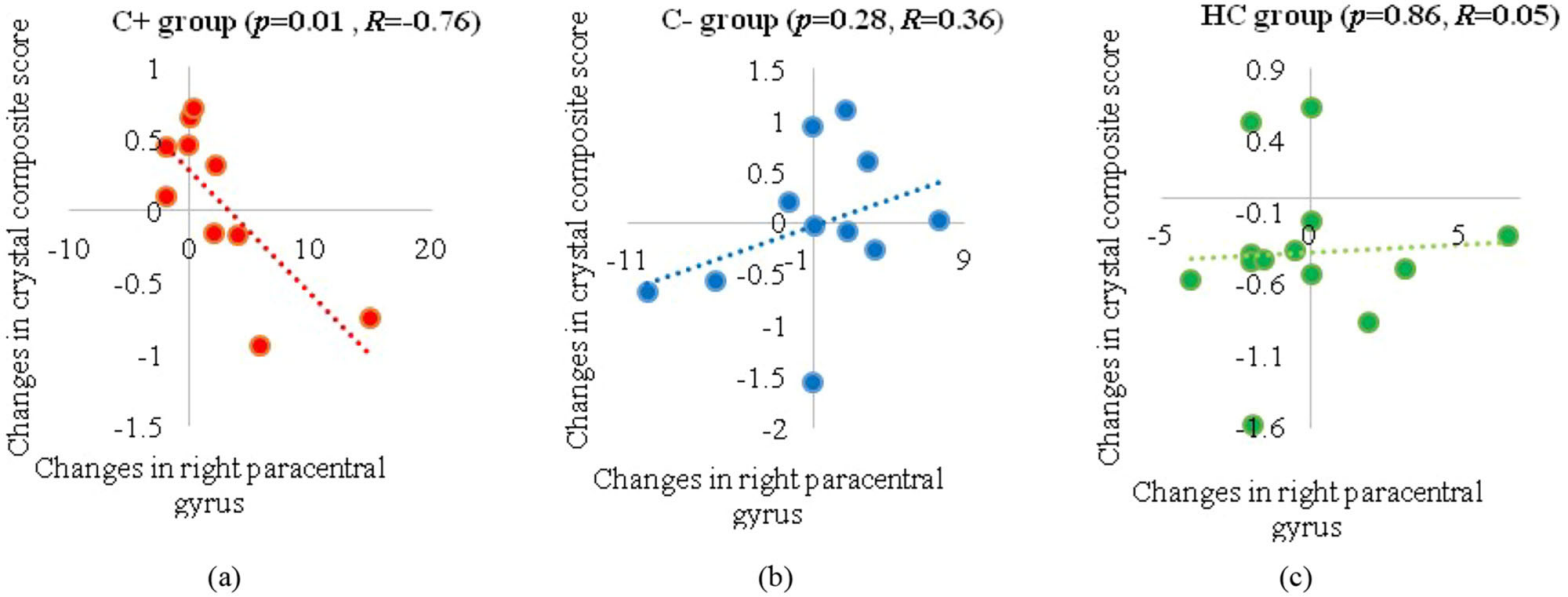
Correlation of longitudinal changes between the right paracentral gyrification values and the crystallized composite scores. (a) chemotherapy (C+) group, (b) no‐chemotherapy (C‐) group, and (c) healthy control (HC) group. *R*: the Pearson's correlation coefficient with significance set at *p* ≤ .05.

The correlation analysis was performed between the significant gyrification alterations within each group over time and the 3 NP composite scores. A significant negative correlation was noted between longitudinal changes in the crystallized composite scores and right paracentral gyrification values in the C+ group (*p *= .01, *R* = −0.76). No significant correlations were noted in the C‐ or the HC group (Figure 4).

## 4 DISCUSSION

We identified altered gyrification in the older long‐term survivors of breast cancer with exposure to chemotherapy. We found the mostly increased gyrification in the chemotherapy‐treated group while only decreased gyrification in the control groups over the 2‐year study interval. In addition, we also found a significant correlation between the increased gyrification and the changes in cognitive testing scores. To the best of our knowledge, this was the first prospective longitudinal study of the effect of chemotherapy on gyrification in older long‐term survivors of breast cancer.

In our previous study of cortical thickness (Daniel et al., 2022), we found cortical thinning in the left hemisphere but no changes in the right hemisphere in the C+ group. In the current study, we observed the gyrification patterns changed in both hemispheres, suggesting that gyrification analysis may be more sensitive to identify early cortical alterations than the cortical thickness parameter. Additionally, the current study revealed significant alterations in gyrification patterns in the control groups, implying an age‐related alteration in all older adults whether or not they had chemotherapy exposure. The cortical thickness study did not show any alteration in cortex in the control groups (Daniel et al., 2022).

We observed an increased gyrification in the right superior temporal gyrus in the C+ group. In contrast, a previous study of breast cancer patients with neoadjuvant chemotherapy showed decreased gyrification in the same region (Zhou et al., 2022). It should be noted that the prior study focused on the acute effects of chemotherapy within 2 months after treatment and assessed the pre‐ and postchemotherapy differences in patients of 29–68 years of age (Zhou et al., 2022). Therefore, the prior study assessed acute changes related to the neurotoxic effects of chemotherapy while our study assessed the chronic chemotherapy‐related neurotoxicity in older long‐term breast cancer survivors. Prior studies of traumatic brain injury showed similar pattern of gyrification alterations in acute and chronic stages, that is, decreased gyrification in patients with mild traumatic brain injury within 3 months of brain injury (Gharehgazlou et al., 2022) and increased gyrification in a cohort with childhood traumatic brain injury after 6–15 years of postinjury (Wilde et al., 2021). Brain changes associated with chemotherapy tend to be subtle and are likely similar to mild traumatic brain injury. One speculation for the increased gyrification relies on the phenomena of neurogenesis (Núñez et al., 2020), in which the brain might expand by increasing gyrification to accommodate newly generated neurons. In addition, the right superior temporal gyrus plays a role in social cognitive function such as auditory and language processing (Bigler et al., 2007). The oral reading recognition score from the NP testing in our study assessed language and auditory skills (Gershon et al., 2014) and was decreased within the C+ group, thus implying the brain structure including the superior temporal gyrus underlying these functions, may be altered. Therefore, we speculate that the increased right superior temporal gyrification might be a compensatory measure to accommodate the newly generated neurons to counter neurotoxicity of chemotherapy (Núñez et al., 2020).

We found increased gyrification in the right medial temporal gyrus in the C+ group, which was in general agreement with a prior study in patients with early stages of dementia (Núñez et al., 2020). Patients with mild cognitive impairment (MCI) and Alzheimer's Dementia (AD) (Núñez et al., 2020) had increased gyrification and atrophy in entorhinal cortex, which was a part of the medial temporal gyrus and was associated with episodic memory (Dickerson & Eichenbaum, 2010). We also found a decreased picture vocabulary testing score in the C+ group, indicating diminished episodic memory. Our findings support the notion that gyrification alteration in the medial temporal lobe may be potentially useful as an imaging biomarker for CRCI and AD in older cancer survivors. The right fusiform gyrus, close to the medial temporal gyrus, also showed increased gyrification in our C+ group. The fusiform gyrus plays an important role in semantic memory such as face recognition (Cai et al., 2015), visual perception (Bokde et al., 2006) and face stimuli (Bokde et al., 2006). Our own prior study noted GM reduction in the right fusiform cortex in the chemotherapy‐treated group (Chen et al., 2018). Overall, our findings implicate the temporal lobe structures as being vulnerable to chemotherapy neurotoxicity.

We found increased gyrification in the paracentral gyrus within the C+ group over time and our findings were consistent with a prior study showing decreased sulcus depth in the paracentral gyrus during the early postchemotherapy phase in breast cancer patients (Zhou et al., 2022). The paracentral gyrus is the medial continuation of the precentral and postcentral regions, which controls motor and sensory innervations of the contralateral lower extremity (Patra et al., 2021). Our findings implicate the paracentral gyrification as a potential neural correlate for CRCI in older long‐term cancer survivors who had chemotherapy treatment many years ago. The increased gyrification in the paracentral gyrus region had a significant negative association with the crystallized composite scores in the C+ group. The crystalized intelligence consisted of picture vocabulary and oral reading recognition based on past learning experiences (Doucet et al., 2022). Nevertheless, the crystalized cognition score was only marginally significant overtime in our C+ group and this score has been known to be resilient to change (Bajpai et al., 2022). More studies in larger samples are needed to confirm the association of the crystalized composite score and the paracentral gyrification changes in the older survivors treated with chemotherapy.

We found decreased gyrification in the left superior parietal lobe in the older long‐term breast cancer survivors with history of chemotherapy treatment. A prior study showed similar findings in a cohort of breast cancer patients shortly after chemotherapy (Zhou et al., 2022). The parietal lobe is important for cognitive function, and atrophy of the superior partial lobe is associated with impairment of working memory, attention, and visuomotor functions (Alahmadi, 2021; Koenigs et al., 2009). Taken together, the diminished left superior parietal gyrification may have occurred shortly after chemotherapy and persisted into long‐term survivorship. Nevertheless, a longitudinal study including a prechemotherapy baseline and long‐term follow‐up is needed to assess the trajectory of gyrification alterations.

The control groups in our study showed only decreased gyrification over time with no increase noted, which was consistent with prior studies of normal aging. For instance, a prior study has shown decreased gyrification in the older population as compared to the younger population (Lamballais et al., 2020). The decreased gyrification in the left lingual and right lateral orbitofrontal gyrus in our C‐ group and in the left postcentral and precuneus in the HC group were in line with previous longitudinal study of healthy aging (Núñez et al., 2020). The underlying neural mechanism for decreased gyrification in the aging studies is not well known (Spalthoff et al., 2018). We speculate that it could be partly due to age‐related brain volume loss, leading to less folding of gyrus thus decreased gyrification during the aging process (Spalthoff et al., 2018).

There were limitations to this study. First, our study cohort was small and there was severe attrition during the 2‐year study interval. We will implement measurements and lessons learned from this study to decrease attrition in our future studies. Specifically, we will work more closely with participants to ensure social and family support; regularly engage them to identify and troubleshoot issues and barriers; help them obtain social services and other resources, send regular reminders about their scheduled visits; send monthly newsletters to update them on new research; arrange for multisession imaging for participants who could not tolerate single‐session MRI scans for any reason (e.g., back pain) to enhance tolerance to neuroimaging; and document reasons for participant withdrawal to further improve retention. Second, our study cohort included mostly non‐Hispanic White women, which decreased the generalizability of our gyrification results to other racial and ethnic groups. Third, though gyrification is a significant surface parameter to assess brain alterations, other surface morphology parameters such as sulcal depth may help confirm brain changes. Further analysis of brain surface parameters is ongoing. Fourth, we found only longitudinal changes over a 2‐year interval but not at TP 1 during the initial enrollment. We believe a larger sample size may help to detect subtle differences among the groups at TP 1. Lastly, Future preclinical research is needed to directly test the true cause‐and‐effect evidence on gyrification alterations and cognition. For instance, a preclinical study in mice may be performed using genetic techniques to directly alter cortical folding (Akinci et al., 2021) and then assess the cognitive function of the mice using the Morris water maze test which is a test of spatial learning for mice (Vorhees & Williams, 2006).

Despite the limitations, this study had merits. This was the first longitudinal study to assess the effect of chemotherapy on gyrification in older long‐term survivors of breast cancer. We contributed novel brain structural and functional information to advance CRCI research in older cancer survivors.

## 5 CONCLUSIONS

We identified altered brain surface gyrification and its association with cognitive function in long‐term breast cancer survivors who had chemotherapy 5–15 years ago. This study implicated gyrification as a possible underlying neural correlate of CRCI in older long‐term survivors of cancer.

## AUTHOR CONTRIBUTIONS

**Ebenezer Daniel**: Writing—review and editing; writing—original draft; visualization; validation; software; formal analysis. **Frank Deng**: Writing—review and editing; validation; formal analysis. **Sunita K. Patel**: Writing—review and editing; validation; formal analysis. **Mina S. Sedrak**: Writing—review and editing; validation; formal analysis. **Heeyoung Kim**: Writing—review and editing; validation; formal analysis; software. **Marianne Razavi**: Validation; writing—review and editing; formal analysis; software. **Lan Sun**: Visualization; writing—review and editing; formal analysis. **James C. Root**: Writing—review and editing; validation; formal analysis. **Tim A. Ahles**: Conceptualization; investigation; funding acquisition; writing—review and editing; methodology; data curation; resources; project administration; formal analysis. **William Dale**: Conceptualization; investigation; funding acquisition; methodology; writing—review and editing; project administration; data curation; resources; formal analysis. **Bihong T. Chen**: Conceptualization; investigation; funding acquisition; methodology; validation; visualization; writing—review and editing; project administration; data curation; supervision; resources; formal analysis.

## CONFLICT OF INTERESTS STATEMENT

The authors had no relevant financial or nonfinancial interests to disclose.

### PEER REVIEW

The peer review history for this article is available at https://publons.com/publon/10.1002/brb3.3634.

## CONSENT TO PARTICIPATE

Informed consent was obtained from all individual participants included in the study.

## Data Availability

The datasets generated during the current study are not publicly available due to lack of relevant public database to deposit the data but are available from the corresponding author on reasonable request.

## References

[brb33634-bib-0001] Akinci, E. , Hamilton, M. C. , Khowpinitchai, B. , & Sherwood, R. I. (2021). Using CRISPR to understand and manipulate gene regulation. Development (Cambridge, England), 148(9), dev182667. (in eng). 33913466 10.1242/dev.182667 PMC8126405

[brb33634-bib-0002] Alahmadi, A. A. S. (2021). Investigating the sub‐regions of the superior parietal cortex using functional magnetic resonance imaging connectivity. Insights into Imaging, 12(1), 47. 33847819 10.1186/s13244-021-00993-9 PMC8044280

[brb33634-bib-0003] Bajpai, S. , Upadhayay, A. D. , Banerjee, J. , Chakrawarthy, A. , Chatterjee, P. , Lee, J. , & Dey, A. B. (2022). Discrepancy in fluid and crystallized intelligence: An early cognitive marker of dementia from the LASI‐DAD cohort. Dementia and Geriatric Cognitive Disorders Extra, 12(1), 51–59. (in eng). 35611146 10.1159/000520879 PMC9082145

[brb33634-bib-0004] Bigler, E. D. , Mortensen, S. , Neeley, E. S. , Ozonoff, S. , Krasny, L. , Johnson, M. , Lu, J. , Provencal, S. L. , McMahon, W. , & Lainhart, J. E. (2007). Superior temporal gyrus, language function, and autism. Developmental Neuropsychology, 31(2), 217–238. (in eng). 17488217 10.1080/87565640701190841

[brb33634-bib-0005] Bokde, A. L. , Lopez‐Bayo, P. , Meindl, T. , Pechler, S. , Born, C. , Faltraco, F. , Teipel, S. J. , Möller, H. J. , & Hampel, H. (2006). Functional connectivity of the fusiform gyrus during a face‐matching task in subjects with mild cognitive impairment. Brain, 129(Pt 5), 1113–1124. (in eng). 16520329 10.1093/brain/awl051

[brb33634-bib-0006] Cai, S. , Chong, T. , Zhang, Y. , Li, J. , von Deneen, K. M. , Ren, J. , Dong, M. , Huang, L. , & Alzheimer's Disease Neuroimaging Initiative . (2015). Altered functional connectivity of fusiform gyrus in subjects with amnestic mild cognitive impairment: A resting‐state fMRI study. Frontiers in Human Neuroscience, 9, 471, (in English) Original Research. 26379534 10.3389/fnhum.2015.00471 PMC4550786

[brb33634-bib-0007] Calvio, L. , Peugeot, M. , Bruns, G. L. , Todd, B. L. , & Feuerstein, M. (2010). Measures of cognitive function and work in occupationally active breast cancer survivors. Journal of Occupational and Environmental Medicine, 52(2), 219–227. 20134340 10.1097/JOM.0b013e3181d0bef7

[brb33634-bib-0008] Cao, B. , Mwangi, B. , Passos, I. C. , Wu, M. J. , Keser, Z. , Zunta‐Soares, G. B. , Xu, D. , Hasan, K. M. , & Soares, J. C. (2017). Lifespan gyrification trajectories of human brain in healthy individuals and patients with major psychiatric disorders. Scientific Reports, 7(1), 511. 28360420 10.1038/s41598-017-00582-1 PMC5428697

[brb33634-bib-0009] Chaudhary, S. , Kumaran, S. S. , Goyal, V. , Kaloiya, G. S. , Kalaivani, M. , Jagannathan, N. R. , Sagar, R. , Mehta, N. , & Srivastava, A. K. (2020). Cortical thickness and gyrification index measuring cognition in Parkinson's disease. International Journal of Neuroscience, 131, 1–10. 32423354 10.1080/00207454.2020.1766459

[brb33634-bib-0010] Chen, B. T. , Conroy, S. K. , Ahles, T. A. , West, J. D. , & Saykin, A. J. (2018). Gray matter density reduction associated with adjuvant chemotherapy in older women with breast cancer. Breast Cancer Research and Treatment, 172(2), 363–370. (in eng). 30088178 10.1007/s10549-018-4911-y PMC6208903

[brb33634-bib-0011] Chen, B. T. , Sethi, S. K. , Jin, T. , Patel, S. K. , Ye, N. , Sun, C. L. , Rockne, R. C. , Haacke, E. M. , Root, J. C. , Saykin, A. J. , Ahles, T. A. , Holodny, A. I. , Prakash, N. , Mortimer, J. , Waisman, J. , Yuan, Y. , Somlo, G. , Li, D. , Yang, R. , … Hurria, A. (2018). Assessing brain volume changes in older women with breast cancer receiving adjuvant chemotherapy: A brain magnetic resonance imaging pilot study. Breast Cancer Research, 20(1), 38. 29720224 10.1186/s13058-018-0965-3 PMC5932862

[brb33634-bib-0012] Daniel, E. , Deng, F. , Patel, S. K. , Sedrak, M. S. , Kim, H. , Razavi, M. , Sun, C. L. , Root, J. C. , Ahles, T. A. , Dale, W. , & Chen, B. T. (2022). Cortical thinning in chemotherapy‐treated older long‐term breast cancer survivors. Brain Imaging and Behavior, 17, 66–76. 36369620 10.1007/s11682-022-00743-5 PMC10156471

[brb33634-bib-0013] de Ruiter, M. B. , Reneman, L. , Boogerd, W. , Veltman, D. J. , Caan, M. , Douaud, G. , Lavini, C. , Linn, S. C. , Boven, E. , van Dam, F. S. , & Schagen, S. B. (2012). Late effects of high‐dose adjuvant chemotherapy on white and gray matter in breast cancer survivors: Converging results from multimodal magnetic resonance imaging. Human Brain Mapping, 33(12), 2971–2983. (in eng). 22095746 10.1002/hbm.21422 PMC6870296

[brb33634-bib-0014] Desikan, R. S. , Ségonne, F. , Fischl, B. , Quinn, B. T. , Dickerson, B. C. , Blacker, D. , Buckner, R. L. , Dale, A. M. , Maguire, R. P. , Hyman, B. T. , Albert, M. S. , & Killiany, R. J. (2006). An automated labeling system for subdividing the human cerebral cortex on MRI scans into gyral based regions of interest. Neuroimage, 31(3), 968–980. (in eng). 16530430 10.1016/j.neuroimage.2006.01.021

[brb33634-bib-0015] Dickerson, B. C. , & Eichenbaum, H. (2010). The episodic memory system: Neurocircuitry and disorders. Neuropsychopharmacology, 35(1), 86–104. 19776728 10.1038/npp.2009.126 PMC2882963

[brb33634-bib-0016] Doucet, G. E. , Hamlin, N. , Kruse, J. A. , Taylor, B. K. , & Poirel, N. (2022). Link between fluid/crystallized intelligence and global/local visual abilities across adulthood. Consciousness and Cognition, 106, 103429. (in eng). 36306570 10.1016/j.concog.2022.103429 PMC10481540

[brb33634-bib-0017] Gershon, R. C. , Cook, K. F. , Mungas, D. , Manly, J. J. , Slotkin, J. , Beaumont, J. L. , & Weintraub, S. (2014). Language measures of the NIH toolbox cognition battery. Journal of the International Neuropsychological Society, 20(6), 642–651. (in eng). 24960128 10.1017/S1355617714000411 PMC4558909

[brb33634-bib-0018] Gershon, R. C. , Wagster, M. V. , Hendrie, H. C. , Fox, N. A. , Cook, K. F. , & Nowinski, C. J. (2013). NIH toolbox for assessment of neurological and behavioral function. Neurology, 80(11), Suppl 3, S2–S6. 23479538 10.1212/WNL.0b013e3182872e5f PMC3662335

[brb33634-bib-0019] Gharehgazlou, A. , Jetly, R. , Rhind, S. G. , Reichelt, A. C. , da Costa, L. , & Dunkley, B. T. (2022). Cortical gyrification morphology in adult males with mild traumatic brain injury. Neurotrauma Report, 3(1), 299–307. (in eng). 10.1089/neur.2021.0032 PMC9438439 36060456

[brb33634-bib-0020] Gregory, M. D. , Kippenhan, J. S. , Dickinson, D. , Carrasco, J. , Mattay, V. S. , Weinberger, D. R. , & Berman, K. F. (2016). Regional variations in brain gyrification are associated with general cognitive ability in humans. Current Biology, 26(10), 1301–1305. 27133866 10.1016/j.cub.2016.03.021 PMC4879055

[brb33634-bib-0021] Hosseini, S. M. H. , Koovakkattu, D. , & Kesler, S. R. (2012). Altered small‐world properties of gray matter networks in breast cancer. BMC Neurology, 12(1), 28. 22632066 10.1186/1471-2377-12-28 PMC3404945

[brb33634-bib-0022] Joly, F. , Giffard, B. , Rigal, O. , de Ruiter, M. B. , Small, B. J. , Dubois, M. , LeFel, J. , Schagen, S. B. , Ahles, T. A. , Wefel, J. S. , Vardy, J. L. , Pancré, V. , Lange, M. , & Castel, H. (2015). Impact of cancer and its treatments on cognitive function: Advances in research from the paris international cognition and cancer task force symposium and update since 2012. Journal of Pain and Symptom Management, 50(6), 830–841. 26344551 10.1016/j.jpainsymman.2015.06.019

[brb33634-bib-0023] Kesler, S. R. , Rao, V. , Ray, W. J. , & Rao, A. (2017). Probability of Alzheimer's disease in breast cancer survivors based on gray‐matter structural network efficiency. Alzheimer's & Dementia: Diagnosis, Assessment & Disease Monitoring, 9, 67–75. 10.1016/j.dadm.2017.10.002 PMC5700833 29201992

[brb33634-bib-0024] Koenigs, M. , Barbey, A. K. , Postle, B. R. , & Grafman, J. (2009). Superior parietal cortex is critical for the manipulation of information in working memory. Journal of Neuroscience, 29(47), 14980–14986. (in eng). 19940193 10.1523/JNEUROSCI.3706-09.2009 PMC2799248

[brb33634-bib-0025] Koppelmans, V. , de Groot, M. , de Ruiter, M. B. , Boogerd, W. , Seynaeve, C. , Vernooij, M. W. , Niessen, W. J. , Schagen, S. B. , & Breteler, M. M. (2014). Global and focal white matter integrity in breast cancer survivors 20 years after adjuvant chemotherapy. Human Brain Mapping, 35(3), 889–899. (in eng). 23281152 10.1002/hbm.22221 PMC6869525

[brb33634-bib-0026] Laird, N. M. , & Ware, J. H. (1982). Random‐effects models for longitudinal data. Biometrics, 38(4), 963–974. (in eng). 7168798

[brb33634-bib-0027] Lamballais, S. , Vinke, E. J. , Vernooij, M. W. , Ikram, M. A. , & Muetzel, R. L. (2020). Cortical gyrification in relation to age and cognition in older adults. Neuroimage, 212, 116637. (in eng). 32081782 10.1016/j.neuroimage.2020.116637

[brb33634-bib-0028] Lepage, C. , Smith, A. M. , Moreau, J. , Barlow‐Krelina, E. , Wallis, N. , Collins, B. , MacKenzie, J. , & Scherling, C. (2014). A prospective study of grey matter and cognitive function alterations in chemotherapy‐treated breast cancer patients. SpringerPlus, 3(1), 444. 25184110 10.1186/2193-1801-3-444 PMC4149682

[brb33634-bib-0029] Li, X. , Chen, H. , Lv, Y. , Chao, H. H. , Gong, L. , Li, C. R. , & Cheng, H. (2018). Diminished gray matter density mediates chemotherapy dosage‐related cognitive impairment in breast cancer patients. Scientific Reports, 8(1), 13801. 30218006 10.1038/s41598-018-32257-w PMC6138678

[brb33634-bib-0030] Liu, T. , Lipnicki, D. M. , Zhu, W. , Tao, D. , Zhang, C. , Cui, Y. , Jin, J. S. , Sachdev, P. S. , & Wen, W. (2012). Cortical gyrification and sulcal spans in early stage Alzheimer's disease. PLoS ONE, 7(2), e31083. (in eng). 22363554 10.1371/journal.pone.0031083 PMC3283590

[brb33634-bib-0031] Luders, E. , Kurth, F. , Mayer, E. , Toga, A. , Narr, K. , & Gaser, C. (2012). The unique brain anatomy of meditation practitioners: Alterations in cortical gyrification. Frontiers in Human Neuroscience, 6, 34, (in English) Original Research. 22393318 10.3389/fnhum.2012.00034 PMC3289949

[brb33634-bib-0032] Lyon, D. E. , Cohen, R. , Chen, H. , Kelly, D. L. , Starkweather, A. , Ahn, H. C. , & Jackson‐Cook, C. K. (2016). The relationship of cognitive performance to concurrent symptoms, cancer‐ and cancer‐treatment‐related variables in women with early‐stage breast cancer: A 2‐year longitudinal study. Journal of Cancer Research and Clinical Oncology, 142(7), 1461–1474. 27102492 10.1007/s00432-016-2163-y PMC4900943

[brb33634-bib-0033] Madan, C. R. (2021). Age‐related decrements in cortical gyrification: Evidence from an accelerated longitudinal dataset. European Journal of Neuroscience, 53(5), 1661–1671. (in eng). 33171528 10.1111/ejn.15039 PMC7979529

[brb33634-bib-0034] Mandelblatt, J. S. , Jacobsen, P. B. , & Ahles, T. (2014). Cognitive effects of cancer systemic therapy: Implications for the care of older patients and survivors. Journal of Clinical Oncology, 32(24), 2617–2626. 25071135 10.1200/JCO.2014.55.1259 PMC4129505

[brb33634-bib-0035] McDonald, B. C. , Conroy, S. K. , Ahles, T. A. , West, J. D. , & Saykin, A. J. (2010). Gray matter reduction associated with systemic chemotherapy for breast cancer: A prospective MRI study. Breast Cancer Research and Treatment, 123(3), 819–828. (in eng). 20690040 10.1007/s10549-010-1088-4 PMC3661415

[brb33634-bib-0036] Miller, K. D. , Nogueira, L. , Devasia, T. , Mariotto, A. B. , Yabroff, K. R. , Jemal, A. , Kramer, J. , & Siegel, R. L. (2022). Cancer treatment and survivorship statistics, 2022. CA: A Cancer Journal for Clinicians, 72(5), 409–436. 35736631 10.3322/caac.21731

[brb33634-bib-0037] Núñez, C. , Callén, A. , Lombardini, F. , Compta, Y. , & Stephan‐Otto, C. (2020). Different cortical gyrification patterns in Alzheimer's disease and impact on memory performance. Annals of Neurology, 88(1), 67–80. (in eng). 32277502 10.1002/ana.25741

[brb33634-bib-0038] Országhová, Z. , Mego, M. , & Chovanec, M. (2021). Long‐term cognitive dysfunction in cancer survivors. Frontiers in Molecular Biosciences, 8, 770413. (in eng). 34970595 10.3389/fmolb.2021.770413 PMC8713760

[brb33634-bib-0039] Patra, A. , Kaur, H. , Chaudhary, P. , Asghar, A. , & Singal, A. (2021). Morphology and morphometry of human paracentral lobule: An anatomical study with its application in neurosurgery. Asian Journal of Neurosurgery, 16(2), 349–354. (in eng). 34268163 10.4103/ajns.AJNS_505_20 PMC8244697

[brb33634-bib-0040] Pendergrass, J. C. , Targum, S. D. , & Harrison, J. E. (2018). Cognitive impairment associated with cancer: A brief review. Innovations in Clinical Neuroscience, 15(1–2), 36–44. (in eng). 29497579 PMC5819720

[brb33634-bib-0041] Perrier, J. , Viard, A. , Levy, C. , Morel, N. , Allouache, D. , Noal, S. , Joly, F. , Eustache, F. , & Giffard, B. (2020). Longitudinal investigation of cognitive deficits in breast cancer patients and their gray matter correlates: Impact of education level. Brain Imaging and Behavior, 14(1), 226–241. (in eng). 30406352 10.1007/s11682-018-9991-0

[brb33634-bib-0042] Schaer, M. , Ottet, M. C. , Scariati, E. , Dukes, D. , Franchini, M. , Eliez, S. , & Glaser, B. (2013). Decreased frontal gyrification correlates with altered connectivity in children with autism. Frontiers in Human Neuroscience, 7, 750, (in English) Original Research. 24265612 10.3389/fnhum.2013.00750 PMC3820980

[brb33634-bib-0043] Siddaway, A. P. , Wood, A. M. , & Taylor, P. J. (2017). The Center for Epidemiologic Studies‐Depression (CES‐D) scale measures a continuum from well‐being to depression: Testing two key predictions of positive clinical psychology. Journal of Affective Disorders, 213, 180–186. (in eng). 28254608 10.1016/j.jad.2017.02.015 PMC6191531

[brb33634-bib-0044] Sousa, H. , Almeida, S. , Bessa, J. , & Pereira, M. G. (2020). The developmental trajectory of cancer‐related cognitive impairment in breast cancer patients: A systematic review of longitudinal neuroimaging studies. Neuropsychology Review, 30(3), 287–309. (in eng). 32607817 10.1007/s11065-020-09441-9

[brb33634-bib-0045] Spalthoff, R. , Gaser, C. , & Nenadić, I. (2018). Altered gyrification in schizophrenia and its relation to other morphometric markers. Schizophrenia Research, 202, 195–202. 30049600 10.1016/j.schres.2018.07.014

[brb33634-bib-0046] Stewart, A. L. , & Ware, J. E. (1992). Measuring functioning and well‐being: The medical outcomes study approach. Duke university Press.

[brb33634-bib-0047] Stouten‐Kemperman, M. M. , de Ruiter, M. B. , Koppelmans, V. , Boogerd, W. , Reneman, L. , & Schagen, S. B. (2015). Neurotoxicity in breast cancer survivors ≥10 years post‐treatment is dependent on treatment type. Brain Imaging and Behavior, 9(2), 275–284. (in eng). 24858488 10.1007/s11682-014-9305-0

[brb33634-bib-0048] van Haren, N. E. , Schnack, H. G. , Cahn, W. , van den Heuvel, M. P. , Lepage, C. , Collins, L. , Evans, A. C. , Hulshoff Pol, H. E. , & Kahn, R. S. (2011). Changes in cortical thickness during the course of illness in schizophrenia. Archives of General Psychiatry, 68(9), 871–880. (in eng). 21893656 10.1001/archgenpsychiatry.2011.88

[brb33634-bib-0049] Vorhees, C. V. , & Williams, M. T. J. N. P. (2006). Morris water maze: Procedures for assessing spatial and related forms of learning and memory. Nature Protocol, 1(2), 848–858. 10.1038/nprot.2006.116 PMC2895266 17406317

[brb33634-bib-0050] Wefel, J. S. , Kesler, S. R. , Noll, K. R. , & Schagen, S. B. (2015). Clinical characteristics, pathophysiology, and management of noncentral nervous system cancer‐related cognitive impairment in adults. CA: A Cancer Journal for Clinicians, 65(2), 123–138. (in eng). 25483452 10.3322/caac.21258 PMC4355212

[brb33634-bib-0051] Weintraub, S. , Dikmen, S. S. , Heaton, R. K. , Tulsky, D. S. , Zelazo, P. D. , Bauer, P. J. , Carlozzi, N. E. , Slotkin, J. , Blitz, D. , Wallner‐Allen, K. , Fox, N. A. , Beaumont, J. L. , Mungas, D. , Nowinski, C. J. , Richler, J. , Deocampo, J. A. , Anderson, J. E. , Manly, J. J. , Borosh, B. , … Gershon, R. C. (2013). Cognition assessment using the NIH Toolbox. Neurology, 80(11), Suppl 3, S54–S64. (in eng). 23479546 10.1212/WNL.0b013e3182872ded PMC3662346

[brb33634-bib-0052] White, T. , Su, S. , Schmidt, M. , Kao, C. Y. , & Sapiro, G. (2010). The development of gyrification in childhood and adolescence. Brain and Cognition, 72(1), 36–45. (in eng). 19942335 10.1016/j.bandc.2009.10.009 PMC2815169

[brb33634-bib-0053] Wilde, E. A. , Merkley, T. L. , Lindsey, H. M. , Bigler, E. D. , Hunter, J. V. , Ewing‐Cobbs, L. , Aitken, M. E. , MacLeod, M. C. , Hanten, G. , Chu, Z. D. , Abildskov, T. J. , Noble‐Haeusslein, L. J. , & Levin, H. S. (2021). Developmental alterations in cortical organization and socialization in adolescents who sustained a traumatic brain injury in early childhood. Journal of Neurotrauma, 38(1), 133–143. (in eng). 32503385 10.1089/neu.2019.6698 PMC7757621

[brb33634-bib-0054] Yang, D. Y. J. , Beam, D. , Pelphrey, K. A. , Abdullahi, S. , & Jou, R. J. (2016). Cortical morphological markers in children with autism: A structural magnetic resonance imaging study of thickness, area, volume, and gyrification. Molecular Autism, 7(1), 11. 26816612 10.1186/s13229-016-0076-x PMC4727390

[brb33634-bib-0055] Youn, H. , Choi, M. , Lee, S. , Kim, D. , Suh, S. , Han, C. E. , & Jeong, H. G. (2021). Decreased cortical thickness and local gyrification in individuals with subjective cognitive impairment. Clinical Psychopharmacology and Neuroscience, 19(4), 640–652. (in eng). 34690119 10.9758/cpn.2021.19.4.640 PMC8553542

[brb33634-bib-0056] Zhou, X. , Tan, Y. , Yu, H. , Liu, J. , Lan, X. , Deng, Y. , Yu, F. , Wang, C. , Chen, J. , Zeng, X. , Liu, D. , & Zhang, J. (2022). Early alterations in cortical morphology after neoadjuvant chemotherapy in breast cancer patients: A longitudinal magnetic resonance imaging study. Human Brain Mapping, 43(15), 4513–4528. (in eng). 35665982 10.1002/hbm.25969 PMC9491291

